# O3.2. BRAIN HYPERACTIVATION DURING MEMORY RETRIEVAL PRECEDES AND PREDICTS CONVERSION TO PSYCHOSIS IN INDIVIDUALS AT CLINICAL HIGH RISK

**DOI:** 10.1093/schbul/sby015.200

**Published:** 2018-04-01

**Authors:** Hengyi Cao, Sarah McEwen, Yoonho Chung, Carrie Bearden, Jean Addington, Bradley Goodyear, Kristin Cadenhead, Barbara Cornblatt, Doreen Olvet, Daniel Mathalon, Thomas McGlashan, Diana Perkins, Aysenil Belger, Larry J Seidman, Ming Tsuang, Theo van Erp, Elaine Walker, Stephan Hamann, Scott Woods, Tyrone Cannon

**Affiliations:** 1 Yale University; 2 University of California, Los Angeles; 3 University of Calgary; 4 University of California, San Diego; 5 Zucker Hillside Hospital; 6 University of California, San Francisco; 7 University of North Carolina; 8 Harvard Medical School, Beth Israel Deaconess Medical Center; 9 University of California, Irvine; 10 Emory University

## Abstract

**Background:**

Memory deficits are a hallmark of psychotic disorders such as schizophrenia. However, whether neural dysfunction underlying these deficits is present prior to onset of illness and potentially predicts conversion to psychosis are unclear. This study aimed to investigate: 1) baseline brain functional alterations during memory processing in subjects at clinical high risk (CHR); 2) whether alterations are more severe in converters compared with non-converters and are thus predictive of psychosis; and 3) associations of these alterations with task performance, baseline symptoms and memory ability.

**Methods:**

A sample of 155 individuals at CHR (including 18 subjects who later converted to psychosis (age 17.22 ± 3.44 years, 10 male) and 137 subjects who did not convert (age 19.01 ± 4.19 years, 81 male)) and 108 healthy controls (age 20.30 ± 4.85 years, 58 male) were drawn from the second phase of the North American Prodrome Longitudinal Study (NAPLS-2) consortium. All participants underwent functional magnetic resonance imaging (fMRI) with a paired-associate memory paradigm, which consisted of one run for encoding and another run for retrieval. During encoding, participants were presented a series of semantically unrelated word pairs and were asked to remember the presented word pair. During retrieval, a pair of words was presented on the screen on each trial and subjects were asked to indicate whether the given word pair had been presented during the encoding session. Active baseline conditions were included in the task for both encoding and retrieval runs.

Data processing was performed for each run, following the standard procedures using the Statistical Parametric Mapping software (SPM12). At individual level, preprocessed images were entered into a general linear model (GLM), generating individual contrast maps (task vs baseline). These contrast maps were further used for a group-level GLM analysis, modeling group, sex, age and site as regressors. Significance was determined using family-wise error (FWE) correction across all voxels in the brain.

The observed activation alterations were further tested for potential associations with task performance, clinical symptoms and/or general memory ability. Task performance was measured using the percentage of correct responses and the mean reaction time during retrieval. Clinical symptoms were evaluated by the summed scores of each domain (positive, negative, disorganization, general) in the Scale of Prodromal Symptoms (SOPS). Memory ability was quantified by the Brief Visuospatial Memory Test- Revised (BVMT-R) and the Hopkins Verbal Learning Test- Revised (HVLT-R) total recall scores.

**Results:**

No significant group differences in activation were found during encoding. However, during retrieval, a significant group effect was observed in five brain regions: left dorsolateral prefrontal cortex (T = 4.75, PFWE = 0.034), left ventrolateral prefrontal cortex (T = 4.99, PFWE = 0.013), left inferior parietal lobule (T = 4.73, PFWE = 0.035), left superior temporal gyrus (T = 5.71, PFWE = 0.001), and right middle temporal gyrus (T = 4.89, PFWE = 0.019). This effect was indicative of greater activation in converters than non-converters and controls and was particularly manifest in unmedicated subjects (P < 0.001). Baseline hyperactivation was correlated with retrieval reaction time during scan in converters (R = 0.61, P = 0.009), and with baseline positive, negative and disorganization symptoms (R > 0.18, P < 0.003) and memory scores (R < -0.15, P < 0.01) in the whole sample.

**Discussion:**

These findings suggest that hyperactivation during memory retrieval may mark processes associated with conversion to psychosis; such measures have potential as biomarkers for psychosis prediction.

